# Amphipoda (Crustacea) from Palau, Micronesia: Families Ampeliscidae, Ampithoidae, Aoridae, Colomastigidae and Cyproideidae

**DOI:** 10.3897/zookeys.193.3109

**Published:** 2012-05-14

**Authors:** Alan A. Myers

**Affiliations:** 1 Department of Biological, Environmental and Earth Sciences, University College Cork, Cork Enterprise Centre, Cork, Ireland

**Keywords:** Amphipoda, taxonomy, new species, Ampeliscidae, Ampithoidae, Aoridae, Colomastigidae, Cyproideidae, Palau, Micronesia

## Abstract

12 species of amphipod in 5 families, collected from shallow reefs in Palau by S. DeGrave during 2002, are reported here. Of these, five species are new to science and *Microdeutopus tridens* Schellenberg (1938) is redescribed and transferred to the genus *Bemlos* Shoemaker (1925). The collection included several additional species in the genera *Amphilochus* Bate, 1862, *Ampithoe* Leach (1814), *Bemlos*, *Byblis* Boeck (1871), *Colomastix* Grube (1861) and *Notopoma* Lowry & Berents (1996), that were either incomplete or juvenile and could therefore not adequately be described. In addition, two new species of *Plumithoe* Barnard & Karaman (1991) are erected from the literature. Other families collected in Palau will be considered in later contributions.

## Introduction

During the 2002 Oxford University Museum Expedition to Palau, amphipods were collected and kindly made available to the author for study by Dr Sammy De Grave. The collection consisted of almost 50 species in 20 families. This first contribution describes species belonging to the families Ampeliscidae, Ampithoidae, Aoridae, Colomastigidae and Cyproideidae. Five species are new to science and are fully described here. In addition, two new species of *Plumithoe* Barnard & Karaman (1991) are erected from the literature.

## Materials and methods

Specimens were dissected in alcohol and mounted on slides in glycerine for study. Drawing was accomplished using a drawing tube attached to a compound microscope. Type material is deposited in the Zoological Collection of the Oxford University Museum of Natural History (OUMNH.ZC).

Abbreviations used in figures. Hd = Head; A1, A2 = antenna 1, 2; Md = mandible, Mxp = maxilliped, G1, G2 = gnathopods 1,2; P3-P7 = pereopods 3-7; Ep 1-3 = epimera 1-3; U1-U3 = uropods 1-3; T = telson; M = male; F = female.

## Systematic section

### Family Ampeliscidae Costa, 1857

#### Ampelisca malakalensis sp. n.

urn:lsid:zoobank.org:act:27622D65-45A2-4FFD-BA7B-3C2C21E37A8C

http://species-id.net/wiki/Ampelisca_malakalensis

Figures 1 –2

##### Type material.

 Holotype male, 6.0 mm. OUMNH.ZC.2002-24-0078, *Halimeda* Flat, flat bottom; from washings of *Halimeda* and sediment, 33 m depth; 07°17.444'N, 134°28.289'E; leg. S. De Grave & P. Colin, 31 May 2002.

##### Etymology.

Named after a harbour near the type locality.

##### Description.

 Based on male holotype. 6.0 mm.

Head. *Head* about one and a half times longer than deep, anteroventral margin oblique. *Eyes*, two pairs each with a cuticular lens; lenses with pigment patches around or near them. *Antenna 1* a little more than half body length; peduncular articles 1 and 2 with numerous bunches of short setae on the posterior margin, peduncular article 2, one and a half times length of article 1; flagellum with 24 articles, reaching end of peduncular article 5 of antenna 2; articles 2–3 with field of long setae. *Antenna 2* equal to body length, peduncular articles 4 and 5 very long, article 5 a little longer than article 4, peduncular article 4 with bunches of short setae on the anterior margin; flagellum with 26 articles. *Mandible* palp article 2 swollen proximally, longer than article 3.

Pereon*. Gnathopod 1* coxa distally rounded without notch. *Gnathopod 2* coxa distally rounded, with small postero-distal notch. *Pereopods 3–4* with dactylus exceeding combined length of carpus and propodus. *Pereopod 5* basis posteroproximal margin with large discrete lobe. *Pereopod 7* basis posterodistal lobe not reaching beyond ischium; propodus inflated, sub-ovoid, dactylus basally expanded.

Pleon. *Epimera 1–2* rounded. *Epimeron 3* with well developed posterodistal spine. *Uropod 3* rami broadly lanceolate, inner margins serrate and with long fine setae. *Telson* a little less than twice as long as broad, cleft to four fifths its length bearing rows of mid-dorsal setae on each side and with distal margins bearing stout setae.

##### Female.

 Unknown.

**Figure 1.**
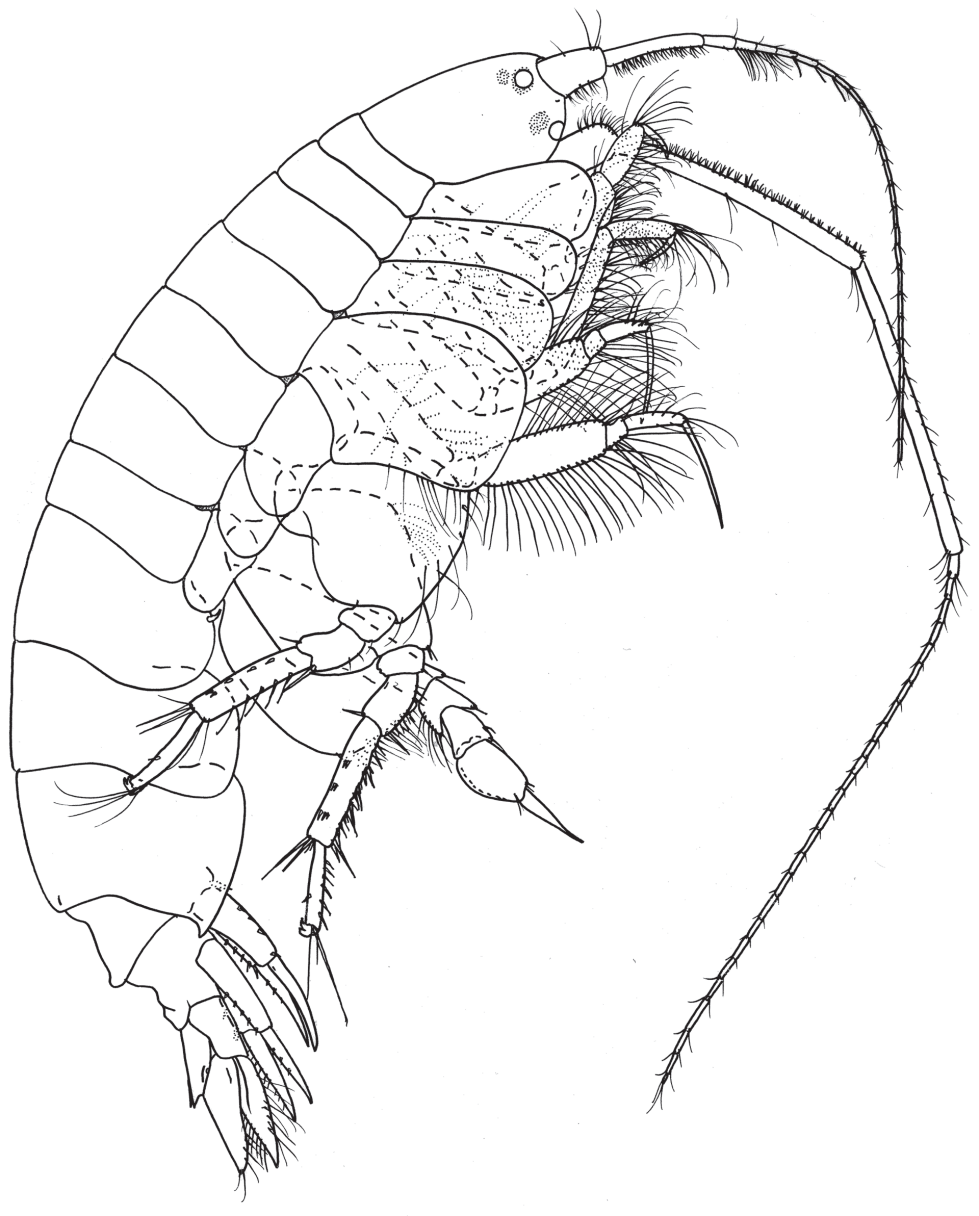
*Ampelisca malakalensis*sp. n., male.

**Figure 2.**
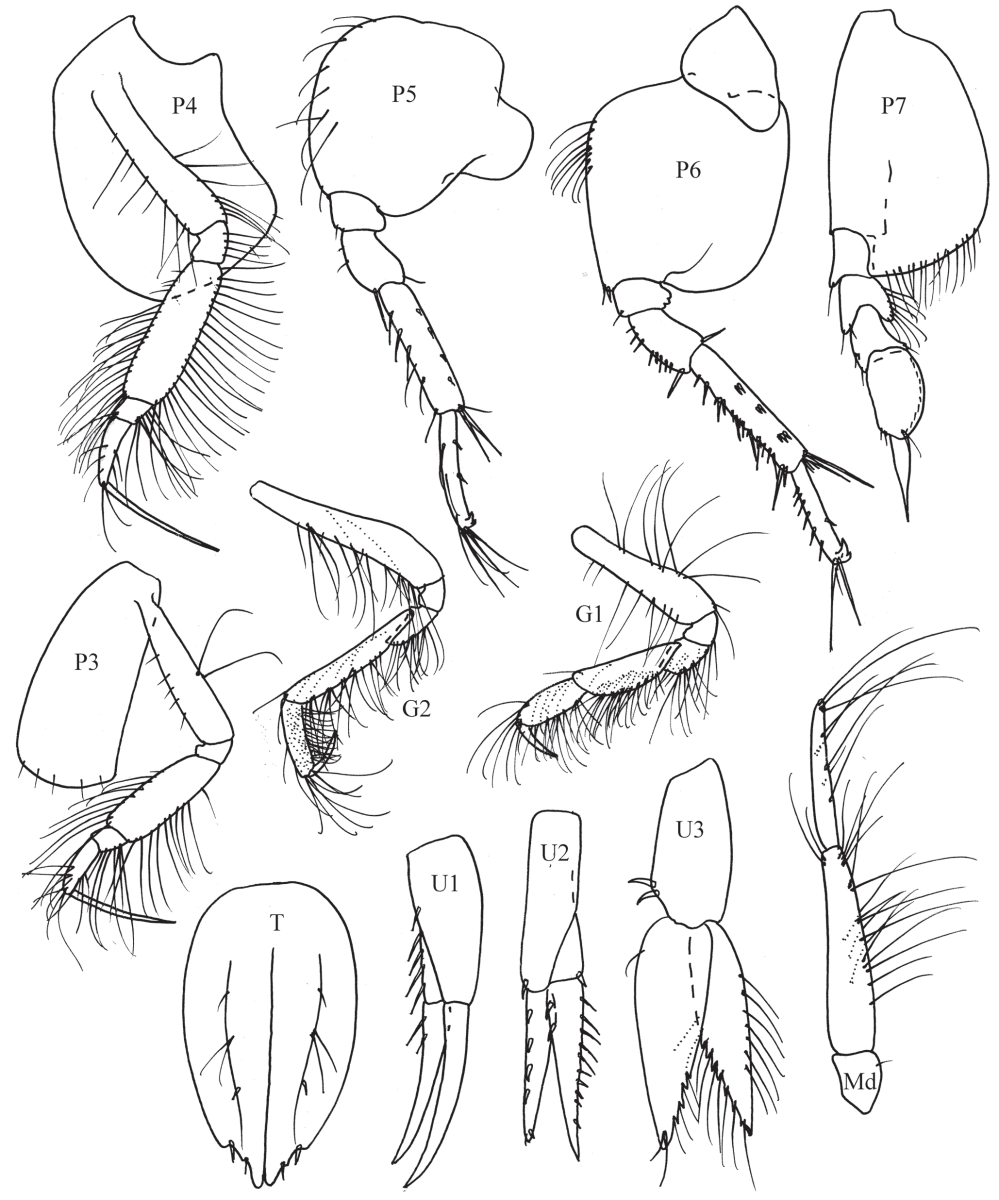
*Ampelisca malakalensis* sp. n., male.

##### Remarks.

*Ampelisca malakalensis* sp. n. resembles *Ampelisca melanesiensis* Myers from Fiji, but that species has a short antenna 1 the flagellum of which does not reach the end of peduncular article 5 of antenna 2. In having antenna 1 flagellum reaching the end of peduncular article 5 of antenna 2, this species is very close to *Ampelisca jiigurru* King from the Great Barrier Reef, but differs from that species in the much more elongated peduncular articles 4 and 5 of antenna 2, the basally swollen mandible palp, the discretely lobed posterior margin of the basis of pereopod 5, the elongate and slender dactylus of pereopod 7, and in the scalloped distal margin of the telsonic lobes.

##### Habitat.

 On soft sediment with *Halimeda*.

##### Distribution.

 Known only from the type locality.

#### Byblis levis sp. n.

urn:lsid:zoobank.org:act:746F29AE-0F26-4989-BA09-A1482D8157A1

http://species-id.net/wiki/Byblis_levis

Figures 3 –4

##### Type material.

 Holotype female, 2.5 mm. OUMNH.ZC.2002-24-0079, Inside Pinchers, bait trap sample, sandy callianasid flat, 3 m depth; 07°20.407'N, 134°25.755'E; leg. S. De Grave & C. Burras, night 27^th^–28^th^ May 2002.

Paratypes. 1 female, OUMNH.ZC.2002-24-0080, Malakal Channel, light trap sample, 2 m deep; 07°17.448'N, 134°28.070'E; leg. S. De Grave & C. Burras, night 21^th^–22^th^ May 2002.

##### Etymology.

Latin levis = lightly armed. In reference to the rather sparse setae of this species compared with other members of the genus

##### Description.

 Based on female holotype. 2.5 mm.

Head. *Head* less than one and a half times as long as deep, anteroventral margin oblique. *Eyes*, two pairs each with a cuticular lens; lenses with strong brown pigment patches around or near them. *Antenna 1* about half body length; peduncular article 2 more than twice times length of article 1; flagellum with 17 articles, reaching well beyond end of peduncular article 5 of antenna 2. *Antenna 2* equal to body length, peduncular article 4 a little longer than article 5, flagellum with 24 articles.

Pereon. *Gnathopods 1–2* coxa distally rounded without notch. *Pereopods 3–4* dactylus shorter than propodus. *Pereopod 5* basis posteroproximal margin with weak lobe. *Pereopod 7* basis posterodistal lobe reaching beyond ischium, weakly scalloped and bearing setae on distal and anterior margins; propodus slender, parallel-sided, dactylus spine-like.

Pleon.*Epimera 1–3* rounded. *Uropod 3* rami broadly lanceolate, inner margins proximally excavate and serrate. *Telson* one and a half times as long as broad, cleft to two fifths its length, distal margins broadly rounded

##### Male.

 Unknown

**Figure 3.**
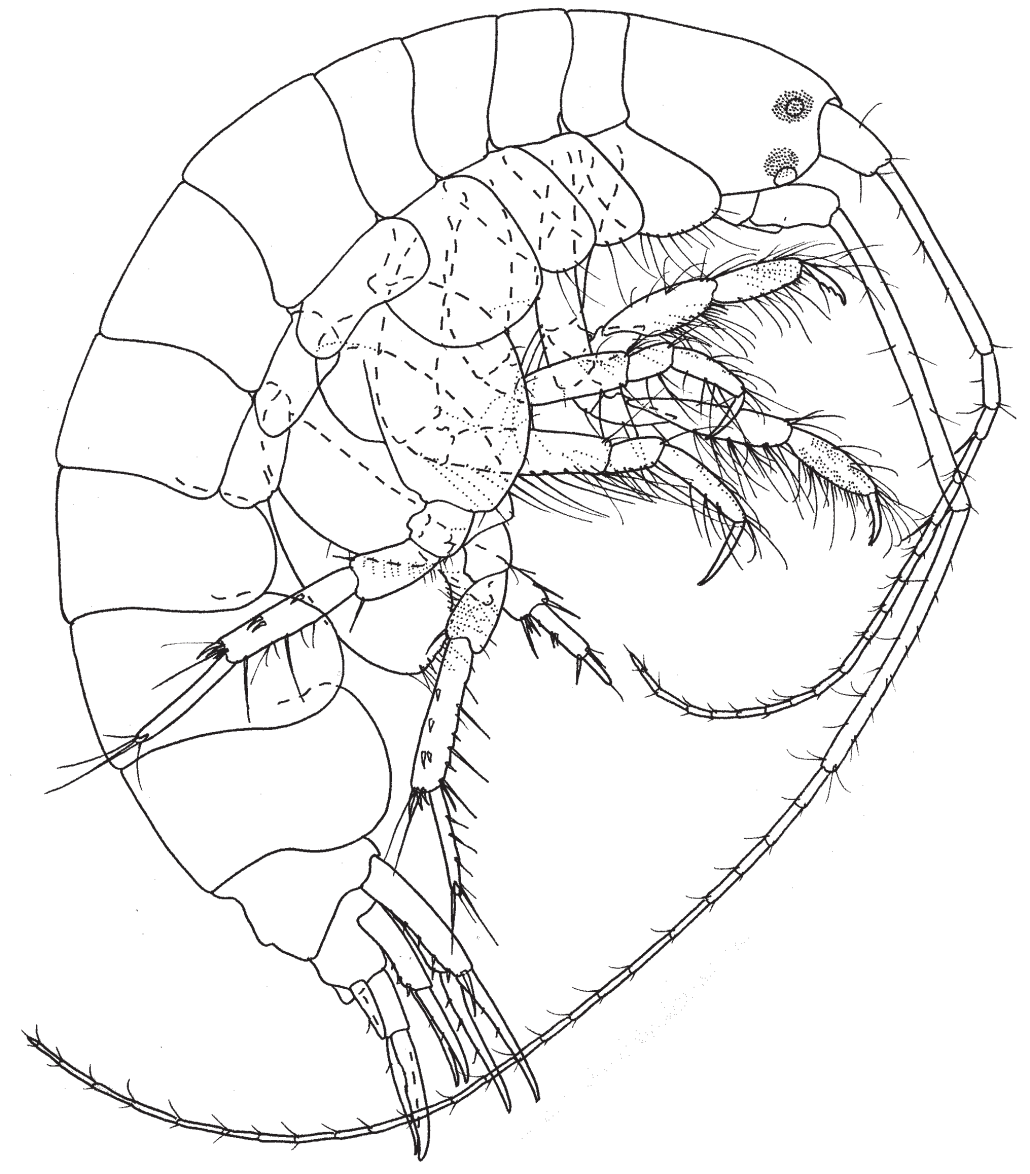
*Byblis levis* sp. n., female.

**Figure 4.**
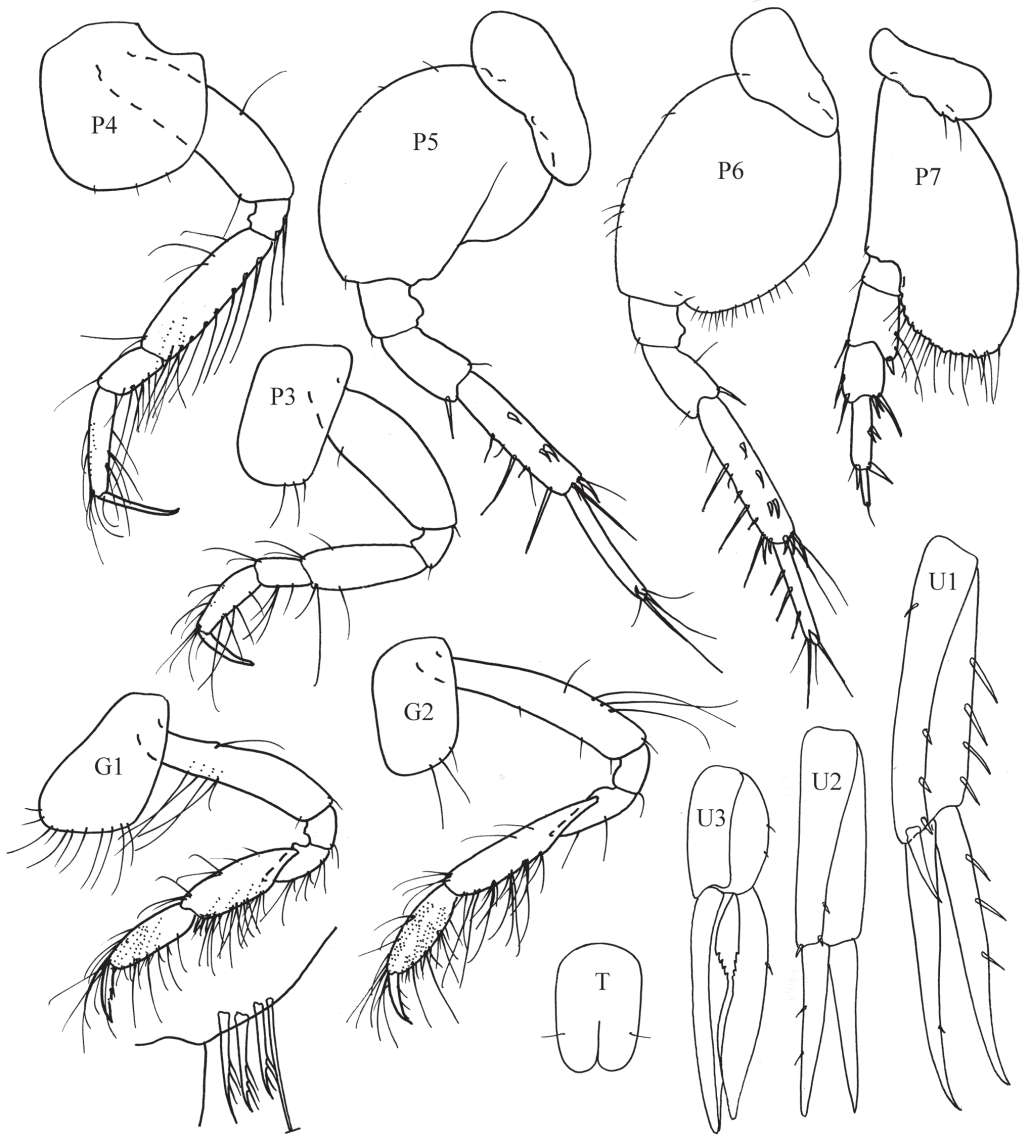
*Byblis levis*sp. n., female.

##### Remarks.

 Species of *Byblis* are only occasionally reported from shallow water, being characteristically found in depths of 20–300 metres. *Byblis* species are rather uniform in design, with character states being found in myriad combinations. This makes it difficult to assign *Byblis* species to groups and therefore difficult to compare a new species with existing species, since each species shares a different suite of characters with different species. The antennal length, distally rounded coxa 1–3, pereopod 7 basis shape and position and number of robust setae on the carpus and propodus, the rather short uropod 2, the slender, elongate uropod 3 rami, and the telson lacking distal setae, when taken in combination distinguish *Byblis levis* sp. n. from all other described species. The possibility cannot be excluded that the material examined my not be fully mature. This could explain the scarcity of setae in the material.

##### Habitat.

 Sand flats in shallow water.

##### Distribution.

 Known only from the type locality.

### Ampithoidae Stebbing, 1899

#### Ampithoe cookana

Peart, 2007

http://species-id.net/wiki/Ampithoe_cookana

Figure 5

Ampithoe cookana Peart 2007b, 13, figs 7-10.–Hughes and Lowry 2009, 154, figs 1–2.

##### Material examined.

 2 males, 10 females, 4 immature, OUMNH.ZC.2002-24-0081, Beluu Lukes Reef, drop off, 15 m depth; from *Melophlus sarasinorum* Thiele, 1899 (Porifera: Ancorinidae); 07°17.530'N, 134°30.870'E; leg. S. De Grave & C. Burras, 2 June 2002; 4 males, 7 females, OUMNH.ZC.2002-24-0082, Ngeritaal Pass, from *Polycarpa captiosa* (Sluiter, 1885) (Ascidiacea: Styelidae), 5 m depth; 07°19.223'N, 134°28.271'E; leg. S. De Grave & C. Burras, 20 May 2002.

**Figure 5.**
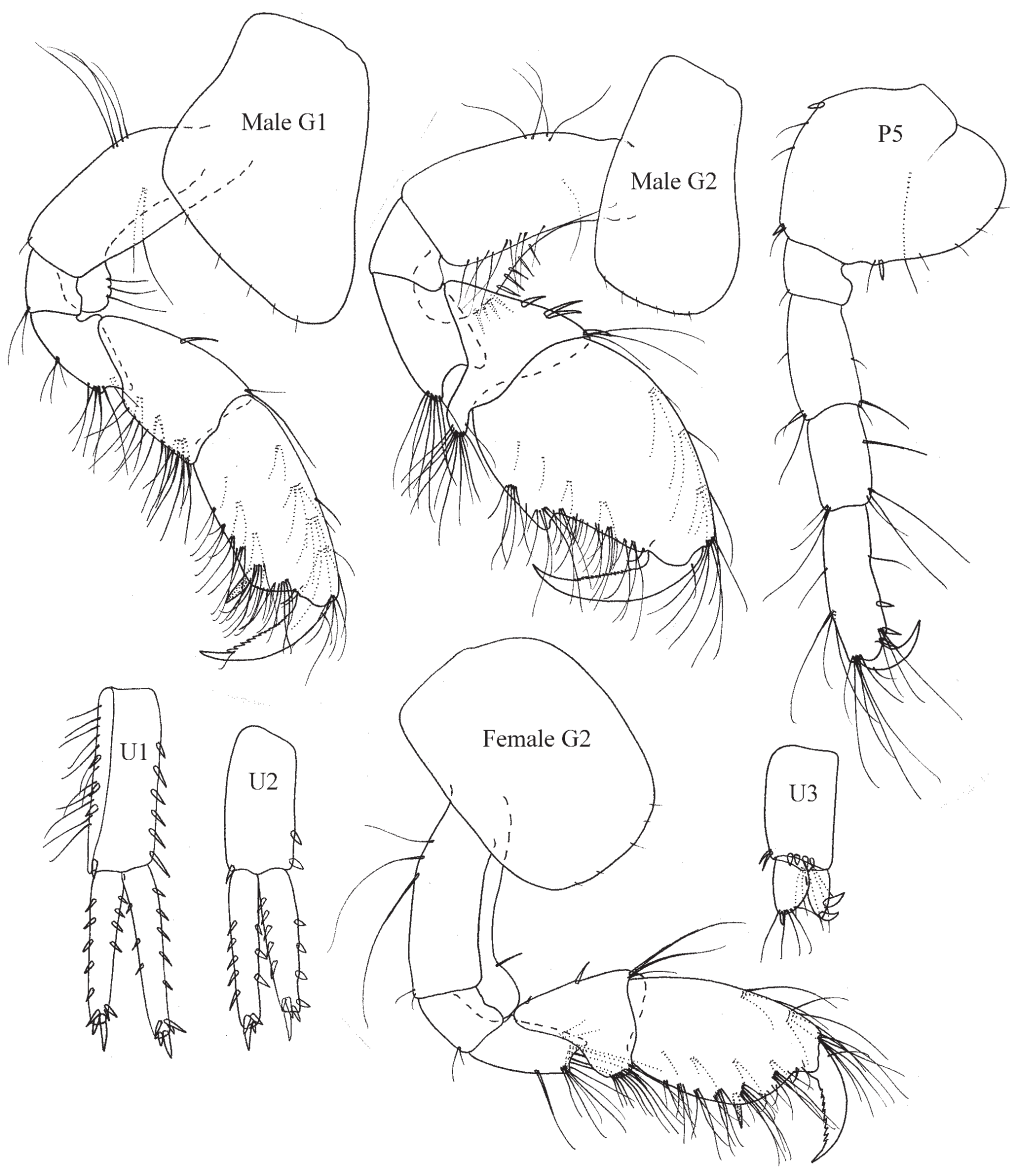
*Ampithoe cookana* Peart, male

##### Remarks.

 Present material agrees well with the original description of the species from the Cook Islands (Peart, 2007b) as well as with material from the Great Barrier Reef, Australia by Hughes and Lowry (2009). Specimens in the current collection from Palau reach a maximum length of 6.5 mm.

##### Distribution.

 Australia (New South Wales, Queensland); Palau.

#### Ampithoe ramondi

Audouin cf

http://species-id.net/wiki/Ampithoe_cf_ramondi

Figure 6

##### Material examined.

 2 males, OUMNH.ZC.2002-24-0083, Ikedluches Reef, outer rubble slope, from unidentified gorgonian with dead base and small amount of algae, 20 m depth; 07°17.987'N, 134°28.756'E; leg. S. De Grave & C. Burras, 25 May 2002; 1 male 1 juvenile, OUMNH.ZC.2002-24-0084, Lighthouse Reef, intertidal collection, consolidated rubble collection; 07°16.658'N, 134°27.670'E; leg. S. De Grave & C. Burras, 26 May 2002.

**Figure 6.**
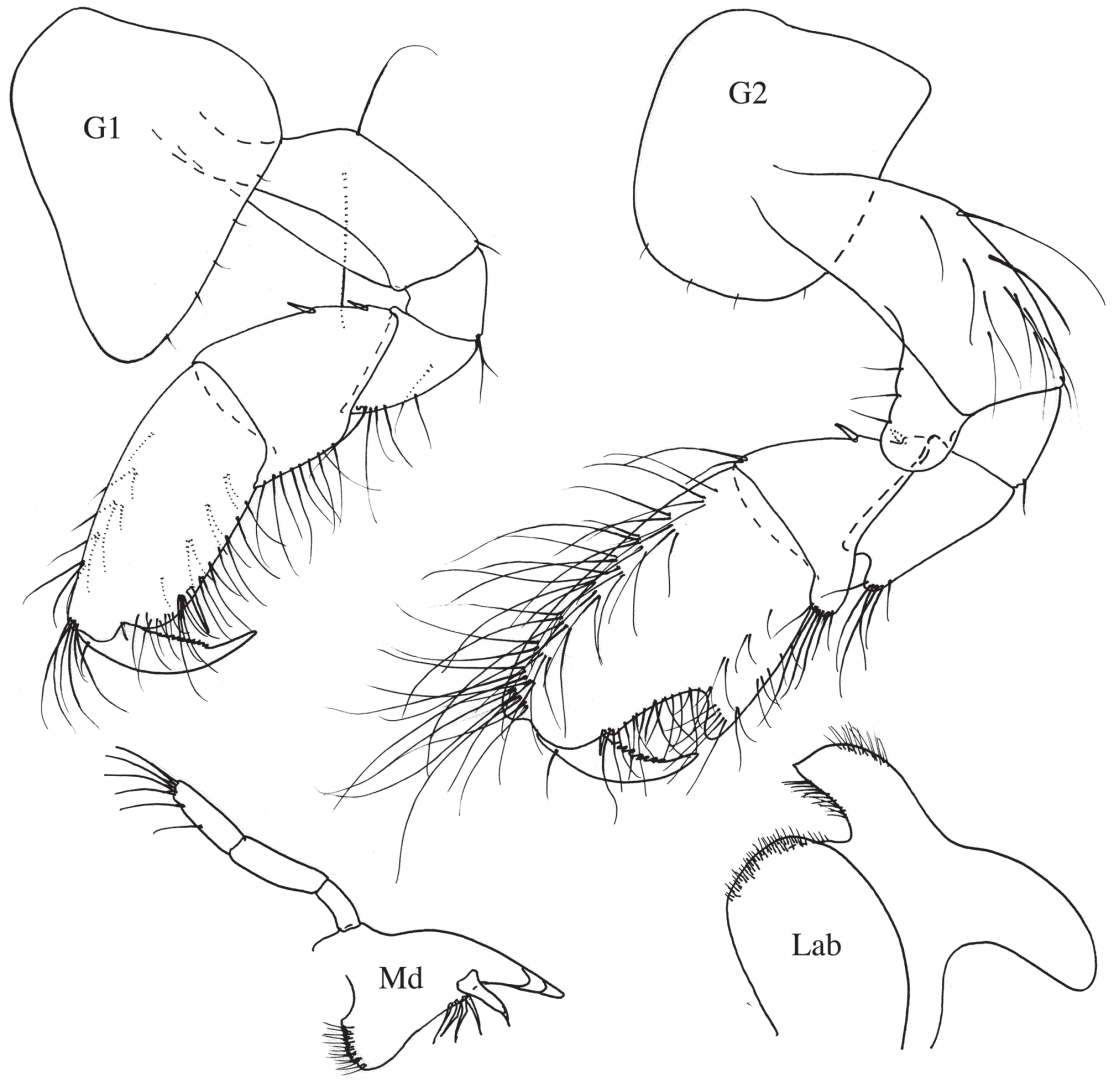
*Ampithoe cf ramondi* Audouin male

##### Remarks.

 As pointed out by Myers (1985), *Ampithoe ramondi* represents a species complex that has yet to be elucidated. Present material agrees very well with material described from Fiji by Myers (1985) under the name *Ampithoe ramondi*. It shows some similarity with *Ampithoe katae* Peart (2007b), from the Great Barrier Reef, but it differs from that species in the more strongly produced posterodistal corner of the male gnathopod 2 merus and carpus and in the more elongate mandible palp article 3. In the latter character state it resembles *Ampithoe cookana* Peart, also from the Great Barrier Reef, but that species has a relatively weakly setiferous propodus anterior margin on the male gnathopod 2. For the moment this material, as well as material described from Fiji by Myers (1985), is simply referred to the *Ampithoe ramondi* complex.

##### Distribution.

 Australia (Queensland, Western Australia); Palau.

#### Cymadusa wistari

Peart, 2007

http://species-id.net/wiki/Cymadusa_wistari

Figure 7

Cymadusa wistari Peart 2007a, 46, figs 37–40.–Hughes and Lowry 2009, 204, figs 31–32.

##### Material examined.

1 male, 13 females**,** OUMNH.ZC.2002-24-0085,Outside Pinchers, from floating algae (*Turbinaria ornata*), leaves on driftline; 07°19.839'N, 134°24.154'E; leg. S. De Grave & C. Burras, 22 May 2002; 3 females,OUMNH.ZC.2002-24-0086,outside Risong, seagrass hand dredge of sediment, 1 m, 07°17.928'N, 134°28.671'E; leg. S. De Grave & C. Burras, 22 May 2002.

**Figure 7.**
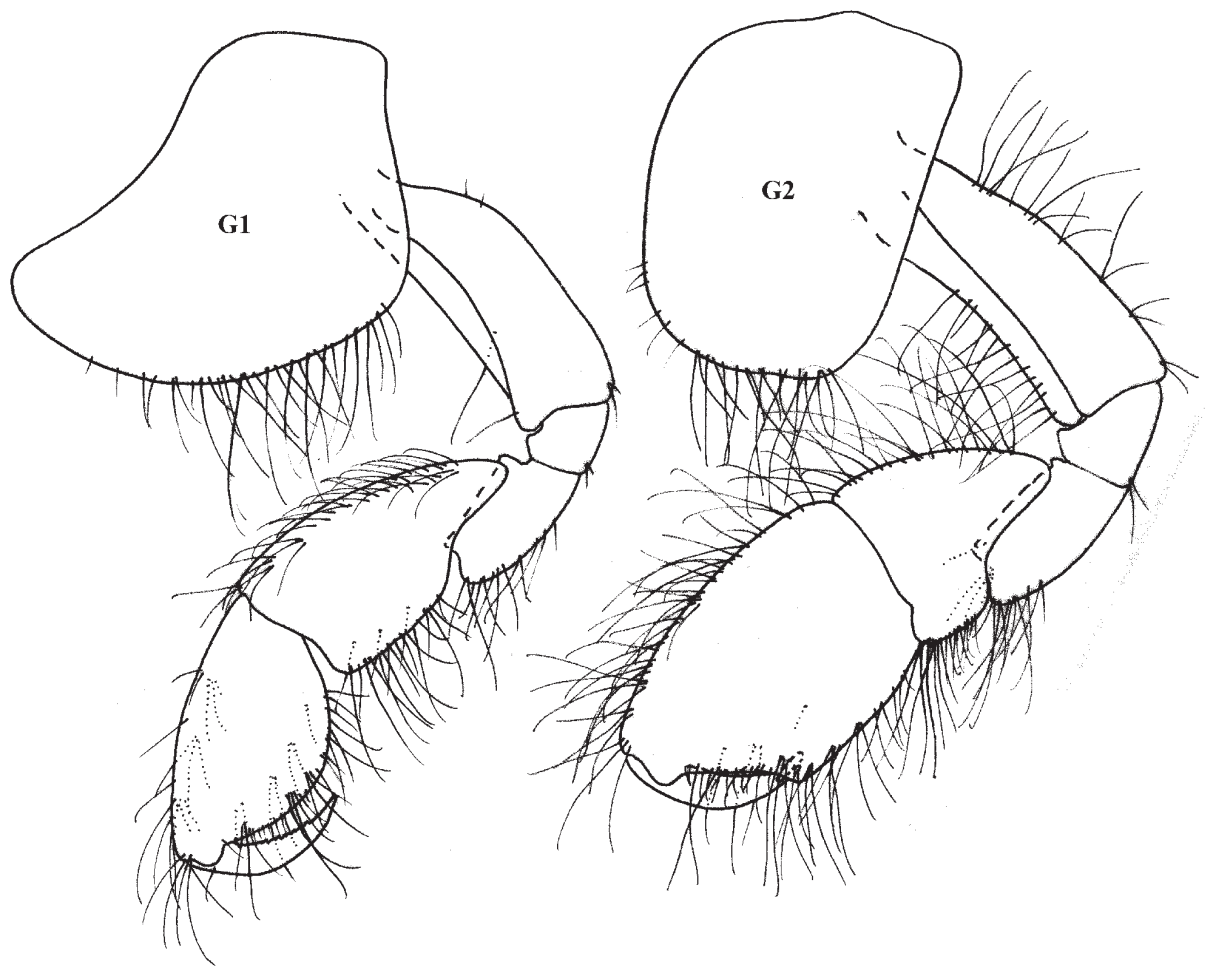
*Cymadusa wistari*Peart, male

##### Remarks.

 Present material agrees well with the description of *Cymadusa wistari* Peart (2007a) from Heron Island. The only apparent difference is the longer setae on the anterior margin of the male gnathopod 2 carpus. It belongs to the ‘*Cymadusa filosa*’ group of species.

##### Distribution.

 Australia (Queensland); Palau.

#### Paragrubia edgari

Hughes & Lowry

http://species-id.net/wiki/Paragrubia_edgari

Figure 8

Paragrubia edgari Hughes & Lowry, 2009, 207, figs 33–34.
Paragrubia vorax .–Myers 1985, 33, figs 24–25 (not *Paragrubia vorax* Chevreux, 1901, 427, figs 50–55.

##### Material examined.

3 males, 4 females, OUMNH.ZC.2002-24-0087, Pkuklim Reef; *Halimeda* clumps on reef rubble, from *Halimeda* (Chlorophyta) washings, 6 m depth; 07°20.542'N, 134°34.023'E; leg. S. De Grave & C. Burras, 29 May 2002.

**Figure 8.**
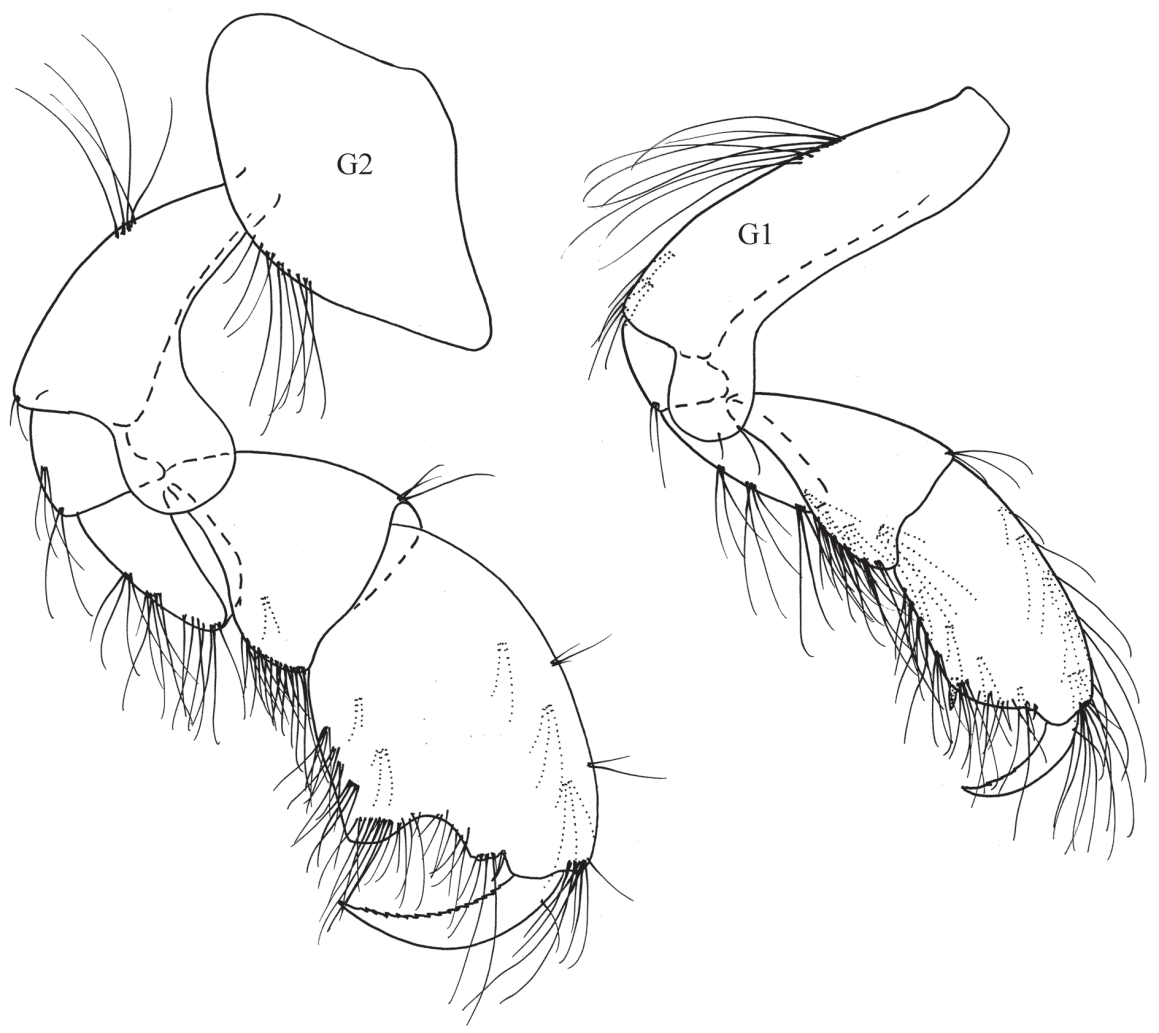
*Paragrubia edgari*Hughes & Lowry, male

##### Remarks.

 Present material agrees well with the description of *Paragrubia edgari* Hughes & Lowry from Lizard Island, Great Barrier Reef. It also agrees with material from Fiji attributed by Myers (1985) to *Paragrubia vorax* Chevreux.

##### Distribution.

 Australia (Queensland); Fiji, Palau.

### Genus Plumithoe Barnard & Karaman (1991)

This genus currently contains three designated species (Poore and Lowry 1997). These are: *Plumithoe hirsuta* (Ledoyer, 1978), *Plumithoe plumicornis* (Ledoyer, 1979) and *Plumithoe quadrimana* (Haswell, 1879).

A fourth species *Plumithoe lata* sp. n. is described here, and two described materials previously allocated to *Plumithoe hirsuta* (Ledoyer), are raised to species status.

#### Plumithoe lata sp. n.

urn:lsid:zoobank.org:act:6D65FAC8-6806-473D-8D91-F540ED96E74A

http://species-id.net/wiki/Plumithoe_lata

Figures 9 –10

##### Type material.

Holotype male 2.2 mm OUMNH.ZC.2002-24-0088 Pkuklim Reef; clumps on reef rubble, from *Halimeda* (Chlorophyta) washings, 6 m depth; 07°20.542'N, 134°34.023'E; leg. S. De Grave & C. Burras, 29 May 2002. Paratypes**.** 2 males, 3 females, 1 imm, collecting data as for holotype.

##### Etymology.

 From the Latin ‘lata’ = wide, in reference to the expanded obtuse elongation of the coxa of the male gnathopod 1.

##### Description.

Based onmale holotype 2.2 mm.

Head. *Head* lateral lobes rounded, anterodistal margin scarcely excavate; eyes medium size. *Antenna 1* a little over half length of body; peduncular articles short; article 3 about half length of article 1; accessory flagellum absent; flagellum more than two times length of peduncle, with 14 articles. Antenna 2 about two thirds length of antenna 1; peduncle short; articles 4 and 5 subequal; articles 3–5 bearing tufts of long setae on the posterior margin; flagellum with 10 articles. *Mandible* palp articles in the ratios (basi-distal) 2:3:3.

Pereon. *Gnathopod 1* coxa anterodistal margin strongly produced, obtuse; basis short, expanded, with large anterodistal flange; propodus longer than carpus, palm evenly rounded; dactylus overlapping palm. *Gnathopod 2* coxa unproduced, deeper than broad; basis robust, expanded, with strongly convex posterior margin and concave anterior margin, moderately produced anterodistal flange; carpus very reduced, cup-shaped, with strongly produced lobe between merus and posterior margin of propodus; propodus elongate, subrectangular, with strong, broad-based posterodistal spine, separated from anterior dactylar lobe by deep triangular excavation; dactylus stout, falciform, slightly overlapping posterodistal spine. *Pereopods 3–4* basis elongate-ovoid; propodus without robust setae; dactylus about half length of propodus. *Pereopods 5–7* with pyrifom basis. *Pereopod 7* scarcely longer than pereopod 6.

Pleon. *Epimeron 3* evenly rounded with minute notch bearing minute seta. *Uropod 1* peduncle longer than rami, with short distoventral spine; outer ramus longer than inner, both rami lacking marginal robust setae. *Uropod 2* peduncle and inner ramus subequal in length; inner ramus longer than outer with one marginal robust seta. *Uropod 3* peduncle longer than rami; rami subequal, outer ramus with 2 recurved robust setae. *Telson* with small telsonic cusps.

**Female** (sexually dimorphic characters). *Gnathopod* 2 basis elongate weakly expanded, lacking strong anterodistal flange; carpus subtriangular.

**Figure 9.**
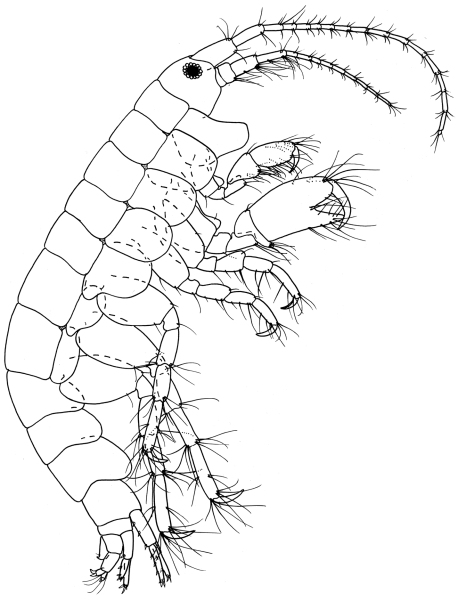
*Plumithoe lata*sp. n., male

**Figure 10.**
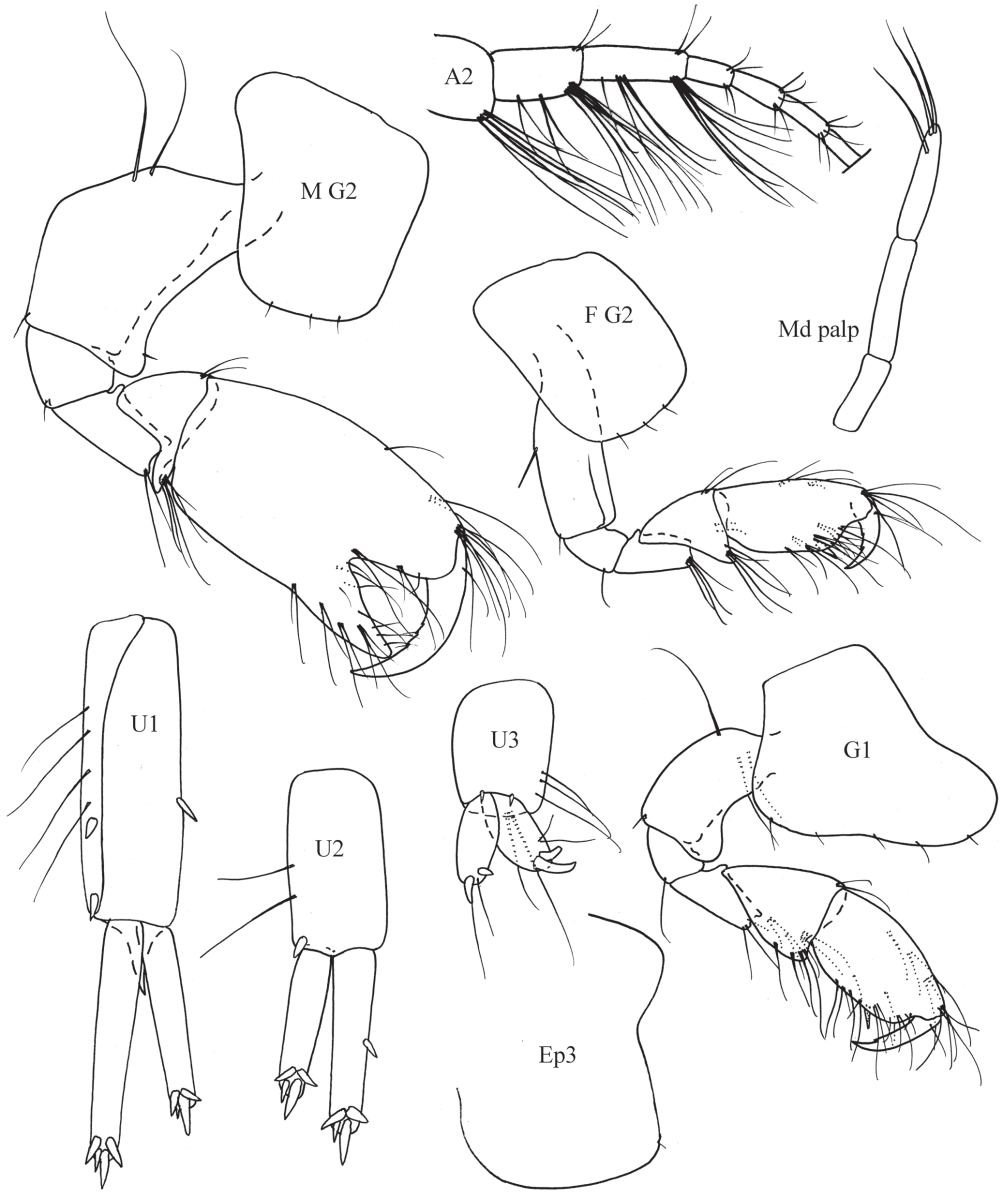
*Plumithoe lata*sp. n., male, female

##### Remarks.

*Plumithoe lata* sp. n.differs from *Plumithoe hirsuta* Ledoyer, from Mauritius and *Plumithoe madagascarienis* sp. n. from Madagascar, in the shape of the male gnathopod 1 coxa, which is expanded both anterodistally and anteroproximally in ***Plumithoe lata***sp. n. but only anterodistally in Madagascan material. It also differs from *Plumithoe hirsuta* Ledoyer in the straight robust spine on the propodus of the male gnathopod 2 (slender and curved in *Plumithoe hirsuta*). *Plumithoe lata* sp. n. also resembles *Plumithoe acuticoxa* sp. n. from Fiji, but Fijian material differs in having a slender basis (as in *Ampithoe pollex* var *hirsutus* from Mauritius) fringed with long setae in the male gnathopod 2 (a unique feature of Fijian material).

##### Habitat.

Amongst *Halimeda*.

##### Distribution.

Known only from the type locality.

#### Plumithoe acuticoxa sp. n.

urn:lsid:zoobank.org:act:E94FBDCE-697C-41FA-A01B-EF965C1C0899

http://species-id.net/wiki/Plumithoe_acuticoxa

Ampithoe hirsuta Myers 1985, 22, figs 13–14.
not Ampithoe pollex var *hirsutus*Ledoyer 1978, 220, fig. 8.

##### Type material.

Holotype male (AM P35333) Makaluva Island, Viti Levu, Fiji, 13 August, 1979, coral debris from reef crest, A. A. Myers.

##### Etymology.

 Named after the shape of the coxa of the male gnathopod 1.

##### Remarks.

 A full description and figures of this species are provided by Myers (1985). *Plumithoe acuticoxa* sp. n. differs from all other described species of *Plumithoe* by its male gnathopod 2 basis fringed with long setae on the anterior margin. In its acute coxa 1, it differs from all described *Plumithoe* species except *Plumithoe quadrimana* (Haswell), but that species lacks a spine on the propodus of the male gnathopod 2. It differs from all other *Plumithoe* species except *Plumithoe hirsuta* Ledoyer in its very short article 3 of the mandible palp but differs from that species in the male coxa 1 and gnathopod 2 basis as described above.

##### Distribution.

 Fiji

#### Plumithoe madagascariensis sp. n.

urn:lsid:zoobank.org:act:34AAABC2-6EFE-4699-937D-2B0D553F498A

http://species-id.net/wiki/Plumithoe_madagascariensis

Ampithoe pollex var *hirsutus*Ledoyer 1982, 122, fig. 41.
not Ampithoe pollex var *hirsutus*Ledoyer 1978, 220, fig. 8.

##### Type material.

 Holotype male (Paris Museum), platier externe de Grand recif de Tulear, station MFE8, MT9, M. Peyrot-Clausade.

##### Etymology.

 Named after the Country in which is situated the type locality.

##### Remarks.

 A description and figures of this species can be found in Ledoyer (1982). *Plumithoe madagascariensis* sp. n. differs from *Plumithoe hirsuta* Ledoyer in its long mandible palp article 3, the more shortened and expanded basis and straight propodal spine of the male gnathopod 2. It differs from *Plumithoe lata* sp. n. in the shape of the male coxa 1 which lacks posterior expansion and has a subtriangular anterodistal projection, in the presence of numerous robust setae on the propdous of pereopod 5 and in the more slender spine on the male gnathopod 2 propodus, It differs from *Plumithoe acuticoxa* sp. n. in its non-acute male coxa 1, in the more robust basis of the male gnathopod 2 that lacks long setae on its anterior margin, and in the long palp article 3 of the mandible palp.

##### Distribution.

 Madagascar.

### Aoridae Stebbing, 1899

#### Bemlos tridens

(Schellenberg) comb. n.

http://species-id.net/wiki/Bemlos_tridens

Figures 11 –12

Microdeutopus tridens Schellenberg 1938, 74, fig. 38.

##### Material examined.

 1 male, 4.0mm, OUMNH.ZC.2002-24-0090,Malakal Harbour channel, rubble tray left for 10 days; 07°19.014'N, 134°27.636'E; leg. S. De Grave & C. Burras, 2 June 2002; 1 male 2 females OUMNH.ZC.2002–24–0091,Omodes intertidal seagrass bed (sparse *Enhalus*) on rubble flat, hand netting; 07°19.439'N, 134°29.231'E; leg. S. De Grave & C. Burras, 23 May 2002.

**Figure 11.**
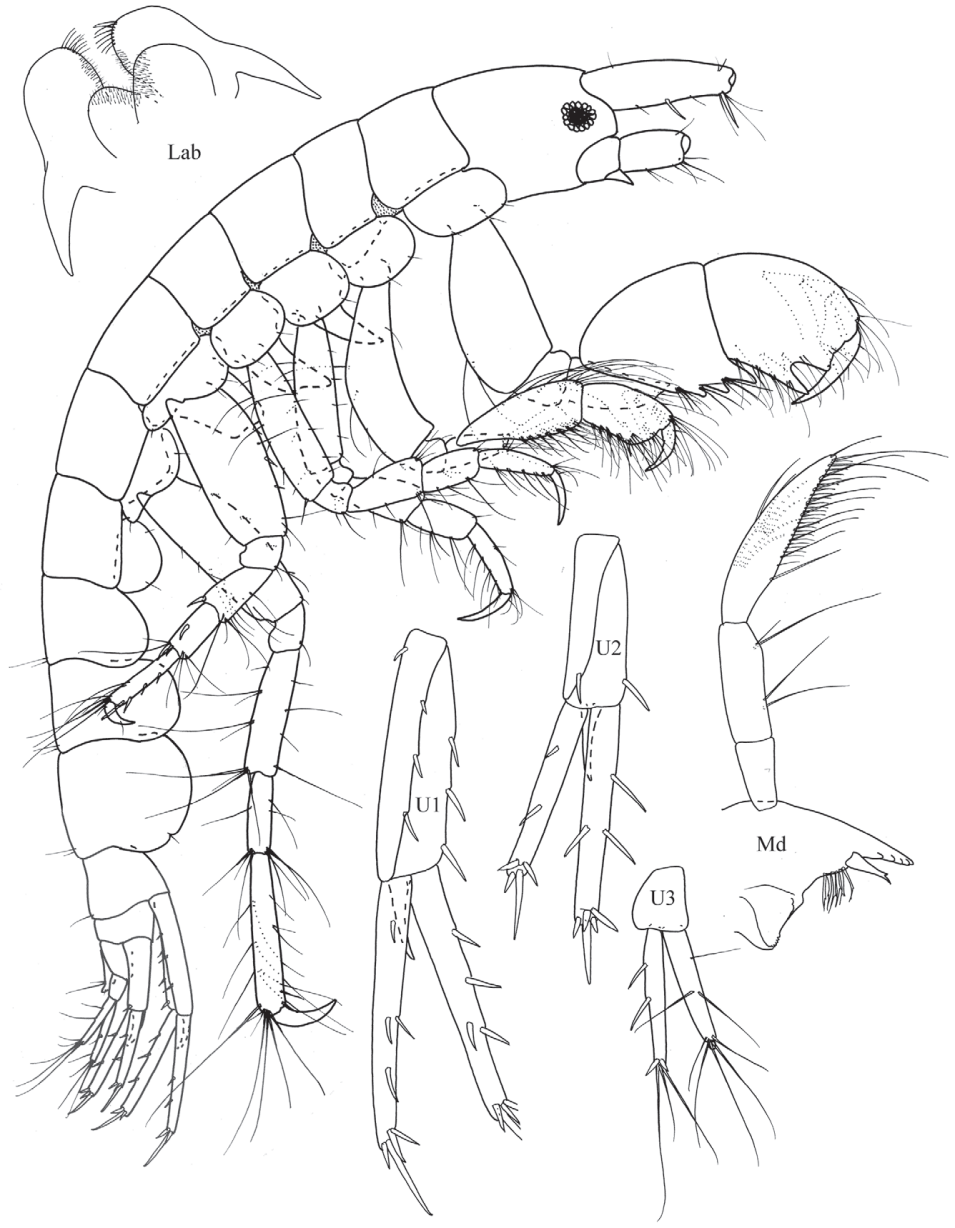
*Bemlos tridens*(Schellenberg) comb. n., male

**Figure 12.**
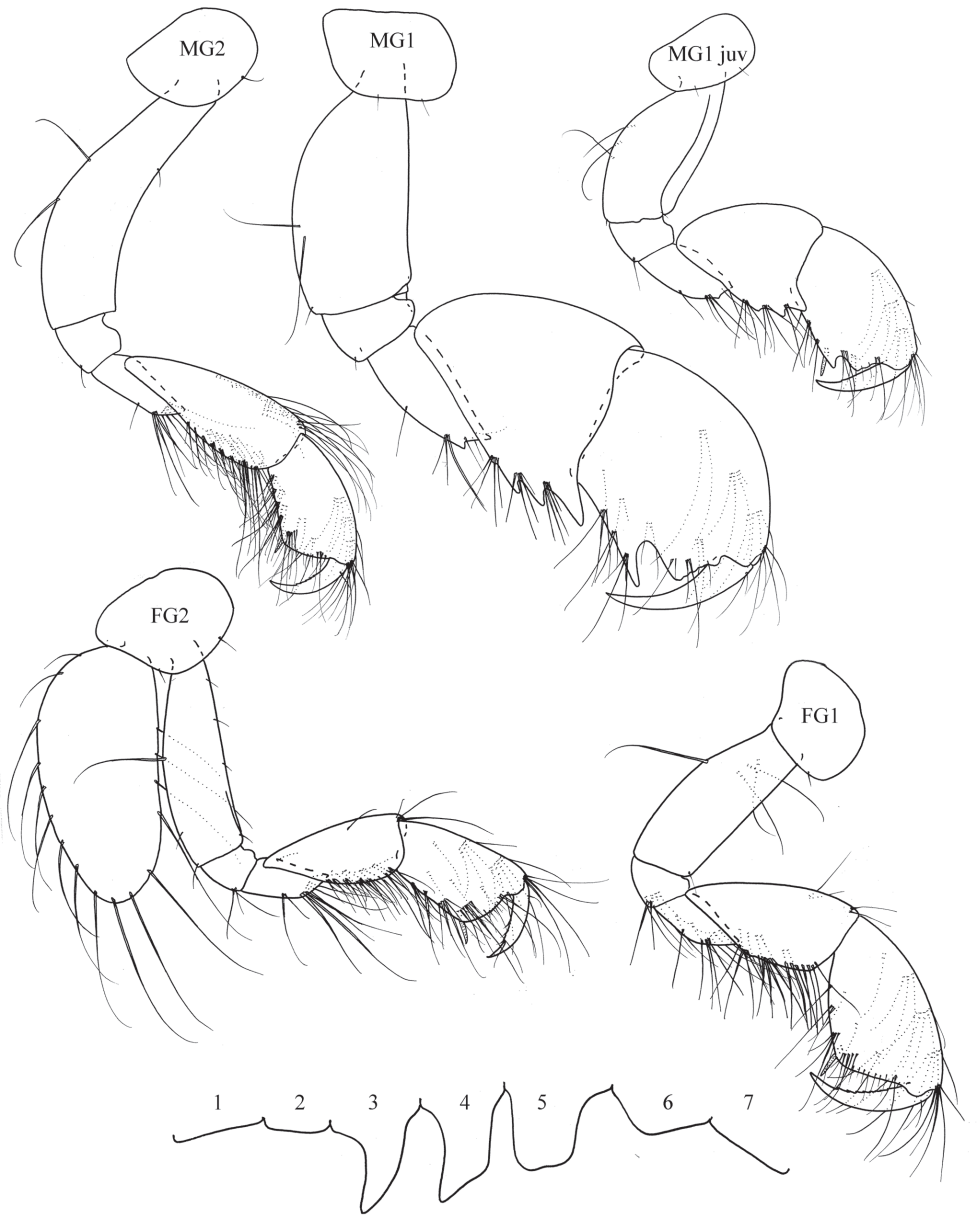
*Bemlos tridens* (Schellenberg), comb. n., male, female

##### Remarks.

 This material agrees very closely with the original description of Schellenberg (1938). The ratios of the mandible palp articles are slightly different at 4:7:11 (Schellenberg gives 2:3:4). Since Schellenberg’s (1938) figures are extremely minimal and somewhat sketchy, and the species has never been reported since, additional figures are presented here.

##### Habitat.

 Coral rubble.

##### Distribution.

 Gilbert Islands (Schellenberg, 1938): Palau.

#### Globosolembos ovatus

Myers, 1985

http://species-id.net/wiki/Globosolembos_ovatus

Lembos (Globosolembos) ovatus Myers 1985a, 354, figs 228–230.
Globosolembos ovatus – Myers 1985b, 47, figs 34–35.– Myers 1989, 66, table 1.– Myers 1995, 33.– Myers 2009, 263, figs 33–34.

##### Material examined.

 1 male, OUMNH.ZC.2002–24–0092, Soint Point Cave, Koror island, light trap sample, no GPS; leg. S. De Grave & C. Burras, night 2^nd^ –3^rd^ June 2002.

##### Distribution.

 Australia; Papua New Guinea; Palau, Vanuatu, Fiji; Western Samoa; Society Islands.

### Colomastigidae Stebbing, 1899

#### Colomastix lecroyae sp. n.

urn:lsid:zoobank.org:act:D6E6C8C8-DCC0-4D27-AE09-27CEFAA7799E

http://species-id.net/wiki/Colomastix_lecroyae

Figures 13 –14

##### Type material.

 Holotype male 2.5 mm, OUMNH.ZC.2002-24-0093, Outside Pinchers, rubble slope, from *Melophlus sarasinorum* Thiele, 1899 (Porifera: Ancorinidae), 10 m depth; 07°20.407'N, 134°25.755'E; leg. S. De Grave & C. Burras, 26 May 2002.

##### Etymology.

 Named for Sara LeCroy for her extensive and invaluable work on this genus of amphipods.

##### Description.

Based on male holotype, 2.5 mm.

Head**.** *Head* longer than peron segment 1; interantennal plate extending far beyond anterodistal angle, anterior margin straight, with ventral spine. Antenna1 1–2 marginal robust setae stout. *Antenna 2* very stout, weakly setiferous.

Pereon**.** *Gnathopod 1* coxa weakly produced; dactylus with two stout distal setae. *Gnathopod 2* coxa evenly rounded; basis grossly swollen distally, anterior margin irregularly scalloped; propodus sub-ovoid, posterior margin with medial spine, palm with small rounded lobe fitting concavity on posterior margin of dactylus. *Pereopods 3–4* with rounded anterodistal lobe. *Pereopods 5–7* basis weakly expanded distally.

Pleon. *Uropod 1 rami* subequal, about two thirds length of peduncle; inner ramus with modified weakly hooked tip. *Uropod 2* rami subequal with each other and with peduncle; inner ramus with weakly curved, acute tip. Uropod 3 rami lanceolate; inner ramus a little shorter than outer ramus and shorter than peduncle. Telson narrowly subtriangular, more than twice as long as broad, distally excavate, and with ventral proximal protrusion.

##### Female.

Unknown

**Figure 13.**
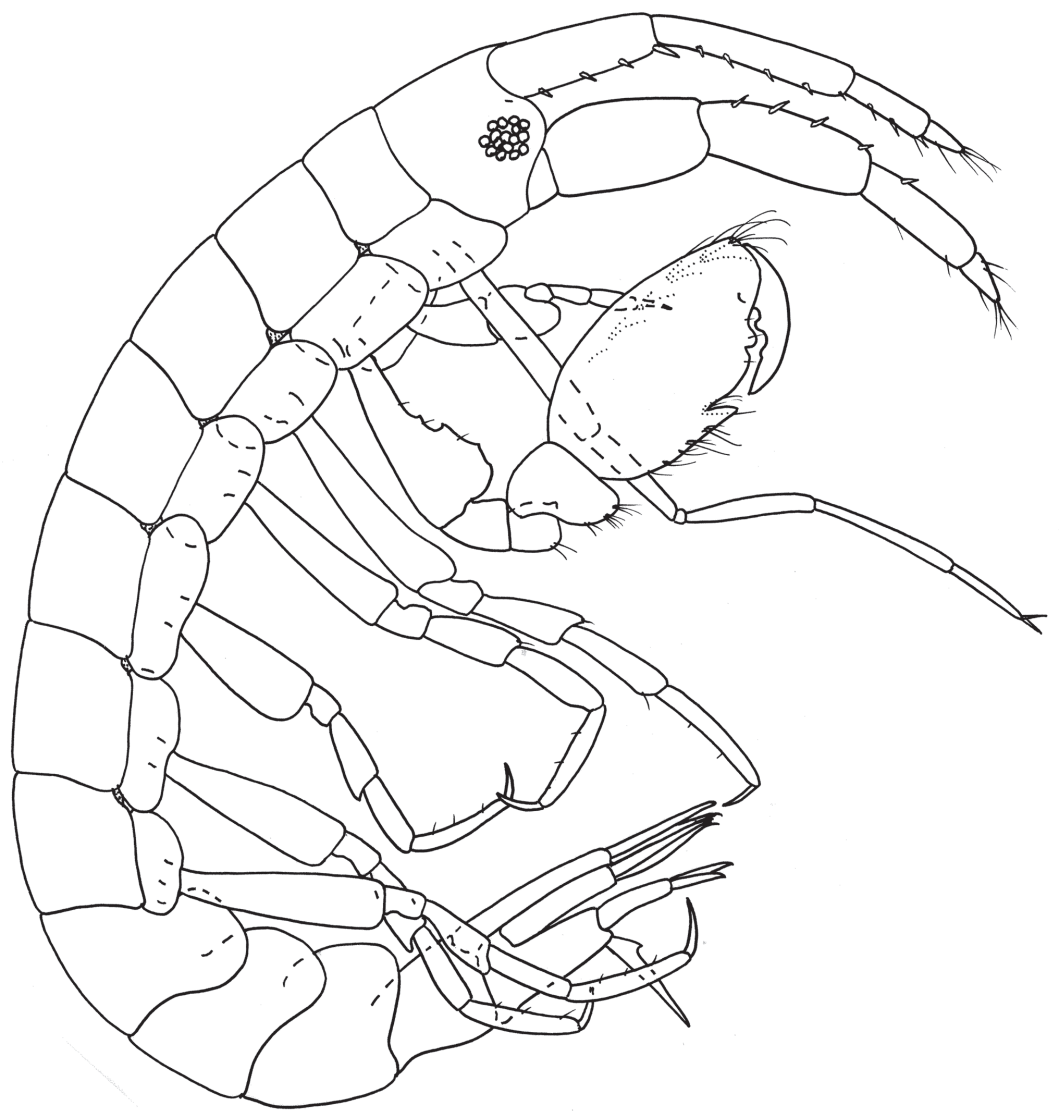
*Colomastix lecroyae*sp n., male

**Figure 14.**
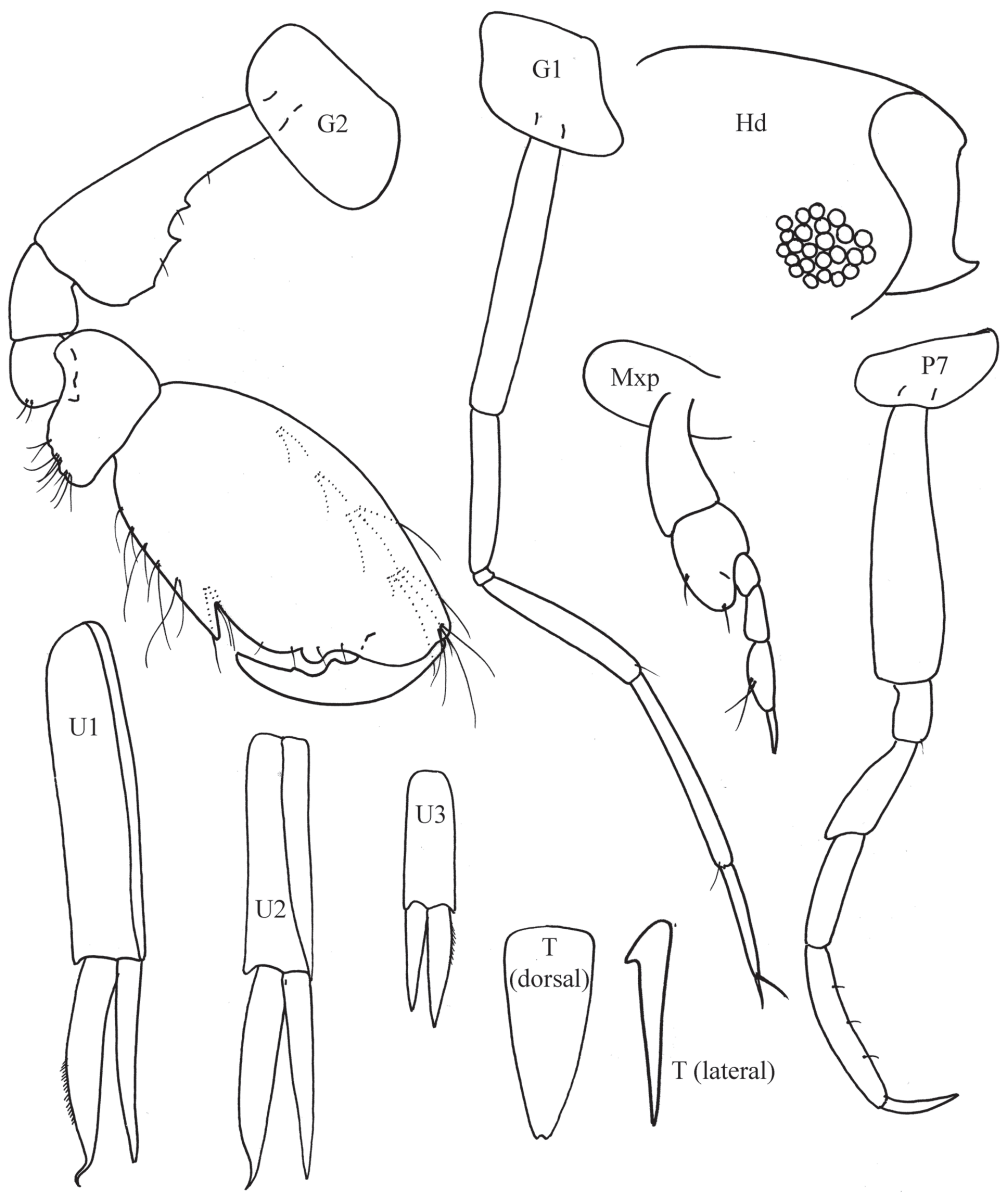
*Colomastix lecroyae* sp. n., male

##### Habitat.

Among sponges**.**

##### Remarks.

In the shape of the male gnathopod 2 this species resembles *Colomastix truncatipes* Ledoyer from Madagascar, but in that species the basis of that appendage has an evenly rounded anterodistal margin, whereas in *Colomastix lecroyae* sp. n. the margin is complexly scalloped. *Colomastix truncatipes* also has very unequal rami on uropod 3, whereas they are almost subequal in *Colomastix lecroyae* sp. n. The single male *Colomastix* sp. described by Ledoyer from New Caledonia is 1.9 mm in total length and appears to be immature. It may be synonymous with *Colomastix lecroyae* sp. n. but has a peculiar character in its bilobed inner lobe of the maxilliped.

##### Distribution.

Known only from the type locality.

#### Colomastix lunalilo

Barnard

http://species-id.net/wiki/Colomastix_lunalilo

Colomastix lunalilo J.L. Barnard 1970: 96–100, figs 51,52.–J. L. Barnard 1971: 55, figs 24, 25.–Ledoyer 1979: 26, fig. 9(2).–Ledoyer 1982: 156–157, fig. 54.–Myers 1985: 56, fig. 41.–Lyons and Myers 1990: 1222, fig. 20.–Muller 1992: 426.–LeCroy 2009: 356–357, figs 5,6.
?Colomastix lunalilo .–Ledoyer 1978: 233, fig. 15(2).
not Colomastix lunalilo .–Hirayama 1990: 21–24, figs 1–3 (= *Colomastix japonica* Bulycheva, 1955).
not Colomastix lunalilo .–Kim and Kim 1987: 9, fig. 8 (= *Colomastix japonica* Bulycheva, 1955).

##### Material examined.

 1 male, OUMNH.ZC.2002–24–0094, Seabear Site, drop off, from rubble (no further details), 16 m depth; 07°16.419'N, 134°31.435'E; leg. S. De Grave & C. Burras, 24 May 2002.

##### Remarks.

The single male specimen collected agrees well with the original description of this species.

##### Distribution.

Hawaii; French Polynesia; Fiji; Australia; ?Mauritius; Madagascar; Red Sea; Palau.

### Cyproideidae Barnard, 1974

#### Cyproidea excavata sp. n.

urn:lsid:zoobank.org:act:C2B98DCF-2197-4097-A721-F9A0EC4654EA

http://species-id.net/wiki/Cyproidea_excavata

Figure 15

##### Type material.

**Holotype** unknown sex, 1.9 mm**.** OUMNH.ZC.2002–24–0095, Pkuklim Reef; *Halimeda* clumps on reef rubble, from *Halimeda* (Chlorophyta) washings, -6 m depth; 07°20.542'N, 134°34.023'E; leg. S. De Grave & C. Burras, 29 May 2002.

**Figure 15.**
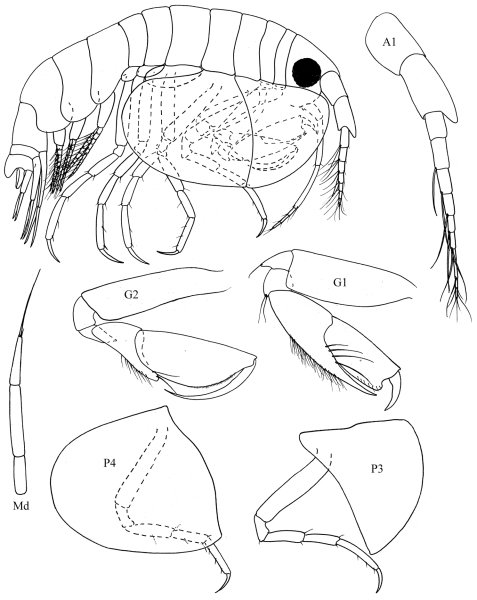
*Cyproidea excavata*sp. n., unknown sex

##### Other material.

1 specimen (unknown sex), dissected. Outside Risong channel, light trap sample,, 50 feet deep; 07°17.903'N, 134°28.544'E; leg. S. De Grave & C. Burras, night 25^th^–26^th^ May 2002.

##### Etymology.

 Named for the excavated palm of gnathopod 2.

##### Description.

 Based on holotype 1.9 mm.

Head. *Eye* very large, round. *Antenna 1* less than one third body length; peduncular articles 1 and 2 subequal in length, article 2 with strong anterodistal spine overreaching half of flagellar article 1; flagellum with 7 articles each bearing a pair of long posterodistal setae. *Antenna 2* slender, longer than antenna 1; peduncular articles 4 and 5 subequal; flagellum shorter than peduncular article 5 with three articles. Mandible palp slender, rod-shaped, article 3 shorter than article 2, narrowing distally with one long and one short distal seta.

Pereon. *Gnathopod 1* coxa vestigial; basis broad; ischium with posterodistal spine; carpus shorter than propodus with long posterodistal acute spine reaching tip of propodus; propodus subrectangular, palm with shallow excavation; dactylus overlapping palm. *Gnathopod 2* basis slender, carpus posterodistal margin with blunt spine extending along one third length of posterior margin of propodus; propodus longer and wider then carpus, subovoid; palm oblique; dactylus elongate, strongly curved, greatly overlapping palm. *Pereopod 3* coxa subtriangular. *Pereopod 4* coxa subovoid. *Pereopods 5–7* subequal in length.

Pleon. Urosomite 3 with dorsodistal hood extending over telson. Uropod 3 rami subequal a little longer than peduncle.

##### Habitat.

 In *Halimeda*

##### Remarks.

*Colomastix excavata* sp. n. appears to be closest to *Colomastix serratipalma* Schellenberg (1938) from New Caledonia, but in that species, on gnathopod 2, the spine on the postero-distal margin of the carpus is very slender throughout its length (broad based in *Colomastix excavata* sp. n.) and the palm is evenly convex. *Cyproidea excavata* sp. n. differs from both *Colomastix liodactyla* Hirayama (1978) and *Colomastix cobia* Azman (2009) in gnathopod 1 which in those species has a serrated palm and spines on the posterior margin of the dactylus. It differs from *Colomastix ornata* Haswell (1880) in the more slender propodus of gnathopod 1, the excavate palm of gnathopod 2, and the subequal rami of uropod 3.

##### Distribution.

Known only from the type locality.

## Supplementary Material

XML Treatment for Ampelisca malakalensis

XML Treatment for Byblis levis

XML Treatment for Ampithoe cookana

XML Treatment for Ampithoe ramondi

XML Treatment for Cymadusa wistari

XML Treatment for Paragrubia edgari

XML Treatment for Plumithoe lata

XML Treatment for Plumithoe acuticoxa

XML Treatment for Plumithoe madagascariensis

XML Treatment for Bemlos tridens

XML Treatment for Globosolembos ovatus

XML Treatment for Colomastix lecroyae

XML Treatment for Colomastix lunalilo

XML Treatment for Cyproidea excavata
